# A Novel Approach to Serving Plant-Based Confectionery—The Employment of Spray Drying in the Production of Carboxymethyl Cellulose-Based Delivery Systems Enriched with *Teucrium montanum* L. Extract

**DOI:** 10.3390/foods13030372

**Published:** 2024-01-23

**Authors:** Ana Mandura Jarić, Laura Haramustek, Laura Nižić Nodilo, Domagoj Vrsaljko, Predrag Petrović, Sunčica Kuzmić, Antun Jozinović, Krunoslav Aladić, Stela Jokić, Danijela Šeremet, Aleksandra Vojvodić Cebin, Draženka Komes

**Affiliations:** 1 Department of Food Engineering, Faculty of Food Technology and Biotechnology, University of Zagreb, Pierotti St 6, 10 000 Zagreb, Croatia; ana.mandura@pbf.unizg.hr (A.M.J.); lauraharamustek@gmail.com (L.H.); dseremet@pbf.hr (D.Š.); avojvodic@pbf.hr (A.V.C.); 2 Institute of Pharmaceutical Technology, Faculty of Pharmacy and Biochemistry, University of Zagreb, Domagojeva St 2, 10 000 Zagreb, Croatia; 3 Department of Thermodynamics, Mechanical Engineering and Energy, Faculty of Chemical Engineering and Technology, University of Zagreb, Savska St 16, 10 000 Zagreb, Croatia; dvrsal@fkit.unizg.hr; 4 Department of Chemical Engineering, Faculty of Technology and Metallurgy, University of Belgrade, Karnegijeva St 4, 11 000 Belgrade, Serbia; ppetrovic@tmf.bg.ac.rs; 5 Forensic Science Centre “Ivan Vučetić” Zagreb, Forensic Science Office, Ilica St 335, 10 000 Zagreb, Croatia; skuzmic@mup.hr; 6 Faculty of Food Technology, Josip Juraj Strossmayer University of Osijek, Franje Kuhača St 20, 31 000 Osijek, Croatia; ajozinovic@ptfos.hr (A.J.); krunoslav.aladic@ptfos.hr (K.A.); stela.jokic@ptfos.hr (S.J.)

**Keywords:** carboxymethyl cellulose, encapsulation, jelly candy, kudzu starch, microparticles, phenylethanoid glycosides

## Abstract

In this study, spray drying was used as a technological solution for the valorization of *Teucrium montanum* extract into carboxymethyl cellulose-based delivery systems (CMC), individually or in combination with collagen, guar gum, gum arabic, and kappa-carrageenan. The results showed that the process yield and morphological properties were positively influenced by the introduction of CMC binary blends. The employment of CMC resulted in a high encapsulation efficiency (77–96%) for all phenylethanoid glycosides (PGs) analyzed. Due to the low wettability of the microparticles, a relatively gradual in vitro release of the PGs was achieved. Infusion of the filling with hydrophilic *T. montanum* extract encapsulated in microparticles with high hydrophobic surface area proved to be a practical route for significant confectionery fortification (5–9 mg PGs per dw serving), ensuring prolonged interaction between the food matrix used and the extract under simulated gastrointestinal conditions. Based on sensory evaluation, the introduction of kudzu starch into the jelly matrix has shown a texture-modifying potential.

## 1. Introduction

Consumer demands on the food and beverage market have never been more challenging than they are today. In addition to basic nutritional value, a valorization of isolated plant-sourced bioactive compounds (BCs), e.g., dietary fibers, vitamins, minerals, bioactive peptides, polyunsaturated fatty acids, polyphenols, etc., in food and nutraceutical products, also promoted the commercialization of herbal extracts as colorants, flavors, or functional ingredients.

Within *Teucrium* (Germanders) genera in *Lamiaceae* family, *T. montanum* has been commonly used in the Mediterranean region, mostly for respiratory diseases [1], to strengthen the immune system [2], to treat digestive complaints [3], as blood purification therapy [4], etc. Reported studies have so far attributed corresponding biological activities to the presence of BCs, with a focus on polyphenols. Supported by significant scientific evidence in terms of disease prevention, anti-aging, and immune system boosting effects, polyphenols have emerged as one of the most attractive antioxidants today [5,6,7]. Among the identified hydroxybenzoic and hydroxycinnamic acids, flavones, and flavone glycosides [8,9,10], phenylethanoid glycosides (PGs) represent the most understudied phenolic group found in *T. montanum* extracts. The latest studies show that PGs are promising neuroprotective [11], anticancer [12], anti-obesity [13], anti-inflammatory [14], and antibacterial [15] agents. The highlighted antiviral properties of PGs, which act as natural inhibitors of SARS-CoV-2 protease, are already being used in China for COVID-19 treatment in the form of a patented TCM formulation—Lianhua Qingwen capsules [16,17].

Despite the recognized biological potential of polyphenols, their relatively low bioavailability, tendency to degrade under various external conditions, e.g., heat, oxygen, enzymes, pH, light, and the possible effects on sensory acceptability pose a major challenge for the food industry. The interplay of their intrinsic properties, i.e., chemical complexity and polarity, with the type of food matrix, particularly affects the final concentration of polyphenols available in the gut, where their actual absorption mainly takes place [18]. To overcome the above-mentioned drawbacks, various encapsulation techniques could serve as an efficient strategy to entrap and protect an active ingredient with a coating material. The spray-drying technique is one of the most studied encapsulation techniques. It ensures the production of fine particles in the range of 1–800 µm with desirable functionalities in terms of stabilizing heat-sensitive, volatile, or unpalatable compounds, improving bioavailability, masking the bittering agent, and extending shelf life [19]. Apart from process parameters, the selection of the carrier material based on weight concentration, molecular weight, linear or branched polymer structure, presence of substitute groups, etc. has a great influence on the quality production of microparticles. The most commonly used carrier materials are carbohydrate polymers such as cellulose and its derivatives, starch and its derivatives, as well as plant exudates, e.g., gum arabic, protein collagen, or whey protein [20,21]. Spray drying has been successfully used for the encapsulation of many heat-labile BC or plant extracts, e.g., cellobiose for carotenoids [22], whey and soy protein for rosemary extract [23], maltodextrin for red wine lees [24], tapioca starch for American elderberry extract [25], maltodextrin, gum arabic, konjac glucomannan, and their binary mixtures for roselle anthocyanins [26], etc.

Due to the tendency in the polymer industry to expand the base of food-grade and renewable biopolymers, various cellulose derivatives, such as carboxymethyl cellulose (CMC), are gaining considerable attention. CMC is a homopolysaccharide whose carboxymethyl groups are attached to some hydroxyl groups of glucopyranose monomers by etherification [27]. Its properties depend on the molecular weight, the number of carboxyl units, and the distribution of carboxyl units within the cellulose backbone. CMC is a generally recognized as safe (GRAS) ingredient with a wide range of applications in the pharmaceutical, food, and packaging industry. It acts as a stabilizer, emulsifier, thickener, and film former due to its good water solubility, moderate oxygen, moisture permeability, and resistance to oils and fats [28,29]. The aforementioned barrier properties are particularly interesting in connection with the functionalization of spray-dried powders, as high wettability and rapid water solubility of microcapsules are not always desirable properties. The valorization of encapsulated delivery systems in food poses a major challenge, as it is difficult to protect the target BCs from severe gastrointestinal conditions while ensuring gradual bioavailability. Therefore, the choice of food matrix (relatively hydrophilic/lyophilic character), the prediction of the interaction between BC and certain macro-components (fats, proteins, and carbohydrates), as well as their stability at certain pH values must be taken into account when considering “food-to-food” fortification.

Considering the scarce literature data for delivering PGs-rich microparticles, the aim of this study was to investigate the suitability of CMC, individually or in combination with hydrolyzed collagen, kappa-carrageenan (κ-carrageenan), guar, and gum arabic for the encapsulation of PGs-rich extract. To this end, we performed an in-depth analysis of the polyphenolic profile of *T. montanum* L., combining nuclear magnetic resonance (NMR) and ultrahigh-performance liquid chromatography coupled with high-resolution mass spectrometry (UHPLC—HRMS/MS), and it proved to be the new outstanding source of PGs [30]. The microparticles produced were first characterized in terms of their physicochemical properties. In particular, process yield, encapsulation efficiency, and loading capacity measured by total hydroxycinnamic acid derivatives (HC) and high-performance liquid chromatography coupled with diode array detector (HPLC-UV-DAD), mean particle size, zeta potential, polydispersity index (PI), wettability, morphology, Fourier transformed infrared spectroscopy (FTIR), and in vitro simulation of digestion were performed. Based on the evaluated results, one sample was selected for incorporation into a confectionery food model. In developing the jelly rolls, the authors were guided by current trends in the global food market, which focus on (i) plant-based ingredients and (ii) the growing popularity of authentic food concepts that offer a unique taste experience, such as mochi cakes [31]. Kudzu (*Pueraria lobata*) is a traditional Chinese herbal plant that has been consumed as a healthy beverage in East Asia for centuries [32]. Kudzu starch is isolated from the kudzu roots and it can be used as a texture modifier and provides pronounced smoothness and pasty properties [33,34]. Five formulations of jelly rolls with a microparticle-enriched filling were prepared using commercial corn starch and kudzu starch as gelatin alternatives. The bioactive characterization, texture, sensory analysis, and finally the release behavior of the HC from the spray-dried delivery system in the chocolate-starch matrix were evaluated. The aim of this study was: (i) to test whether there is a positive or negative influence of the introduced binary mixtures compared to CMC alone on the process yield, physicochemical properties, encapsulation efficiency, and simulated in vitro release of the investigated polyphenols, (ii) to determine the potential influence of the corn-to-kudzu starch ratio on sensory quality and texture profile, and (iii) to confirm the main benefit of encapsulation use in terms of improving the prolonged release of the targeted polyphenols from the delivery system.

## 2. Materials and Methods

### 2.1. Materials

#### 2.1.1. Extract Production and Spray-Drying Encapsulation

Mountain Germander (*T. montanum* L.) was purchased from the local supplier (Ljekovito bilje j.d.o.o, Zadar, Croatia). The plant was collected in Adriatic region of Croatia in August 2020 (municipality of Kistanje, Šibenik-Knin County, Croatia). Voucher specimen (ID: 75518) was deposited in the Herbarium Croaticum (Faculty of Science, Zagreb, Croatia). After sorting, milling, and sieving, areal parts of plant (<450 μm) were used in all experiments. Carboxymethyl cellulose (Biosynth s.r.o. Bratislava, Slovakia), hydrolyzed collagen (Biosynth s.r.o. Bratislava, Slovakia), gum arabic (Alfa Aesar, Kandel, Germany), hydrolyzed guar gum (Palco Nutrifit d.o.o., Velika Gorica, Croatia), and kappa-carrageenan (Gelymar, Puerto Montt, Chile) were used as polymer carriers for extract encapsulation.

#### 2.1.2. Development of Jelly Rolls

Commercial corn starch (Dr. Oetker, Jánossomorja, Hungary), kudzu starch (Clearspring Organic Japanese Kuzu, London, UK), lyophilized raspberries (Dennree, Töpen, Germany), glucose syrup (DE = 42, Fractal, Csömör, Hungary), cold-pressed coconut oil (Ekoplaza, Veghel, Netherlands), and shea butter (Agava Karin Lang, Bremen, Germany) were purchased in specialized store. Dark chocolate (52% of cocoa solids), natural red colorant, and natural raspberry aroma were generously donated from confectionery industry Kraš d.d. (Zagreb, Croatia). Glycerol (85%, *v*/*v*) was purchased in pharmaceutical store, whereas citric acid (Podravka, Koprivnica, Croatia) was bought in the local store.

#### 2.1.3. Chemicals and Reagents

All chemicals and reagents used in the experiments were of per analysis and HPLC grade. Folin–Ciocalteu reagent, potassium chloride, sodium hydrogencarbonate, potassium hydrogenphosphate, magnesium chloride hexahydrate, sodium chloride, ammonium carbonate, sodium hydroxide, and petroleter were supplied from Kemika d.d. (Zagreb, Croatia). Anhydrous sodium carbonate and sodium nitrite were acquired from Gram-Mol d.o.o. (Zagreb, Croatia). Sodium molybdate (>98%) was supplied from abcr Gmbh (Karlsruhe, Germany). Methanol, acetonitrile, concentrated hydrochloric acid (37%, *v*/*v*), sodium hydroxide, and formic acid were purchased from Carlo Erba Reagents (Val de Reuil, France). Secondary analytical standards, i.e., echinacoside (>98%) and verbascoside (>98%), were obtained from Biosynth s.r.o. (Bratislava, Slovakia), whereas caffeine was supplied from Fluka (Taufkirschen, Germany). Theobromine (min. 99%), human salivary α–amylase XII-A (Enzyme Commission number: 3.2.1.1.; 1149 U mg^−1^ protein), pepsin from porcine gastric mucosa (474 U mg^−1^ protein), pancreatin from porcine pancreas (4×USP), and bile bovine salts (unfractionated) were provided from Sigma Aldrich (St. Louis, MO, USA).

### 2.2. Bioactive Characterization of Extract

#### 2.2.1. Preparation of Polyphenolic Extract

According to the results of previously reported optimization study on *T. montanum* [35], conventional extraction technique was employed for the preparation of water extract (1 g:100 mL ratio of mass and solvent, 100 °C, 30 min). Filtered extract was tenfold concentrated on rotary evaporator (RV 8, IKA, Staufen, Germany).

#### 2.2.2. Determination of Total Hydroxycinnamic Acid Derivatives (HC)

For determination of encapsulation efficiency (EE) and loading capacity (LC), as well as the content of HC incorporated in formulated microparticles and candies, spectrophotometric method (GenesysTM 10S UV-VIS, Thermo Fisher Scientific, Waltham, MA, USA) was applied [36]. The results were expressed in % for EE, or in mg of echinacoside equivalents per g of dry matter of plant/powder or candy sample (mg eq ech g^−1^ dw).

#### 2.2.3. HPLC-UV-DAD Quantification of Phenylethanoid Glycosides (PGs)

Since the elucidation and quantification of phenolic compounds in previous studies showed predominant presence of hydroxycinnamic acids derivatives in *T. montanum* extract [30], i.e., phenylethanoid glycosides (PGs), determination of HC as well as HPLC-UV-DAD analysis of echinacoside, stachysosyide A, teupolioside, poliumoside, and verbascoside, were of the main interest in the evaluation of encapsulation efficiency and the bioactive enrichment of jelly rolls. Results for teupolioside, stachysoside A, and poliumoside were expressed in mg of echinacoside equivalents per gram of dry matter of plant/powder or candy sample (mg eq ech g^−1^ dw).

### 2.3. Spray-Drying Encapsulation of Extract

The polymer solutions were prepared by weighing (A&D Instruments technical balance, Abingdon, United Kingdom) one (100%) or two selected polymer carriers in combination (75%:25%) to obtain 0.8% (*w*/*w*) of final polymer content in the concentrated extract solution. CMC, hydrolyzed collagen (CLG), κ-carrageenan (CAR), gum arabic (GA), and hydrolyzed guar gum (GG) were used as carrier materials. The solutions were homogenized on a magnetic stirrer for 24 h until they were completely dissolved (SMHS-6, Witeg Labortehnik GmbH, Wertheim, Germany).

Spray drying of the prepared polymer solutions was carried out using a laboratory device Büchi Mini Spray Dryer B-290 (Flawil, Switzerland). Experimental conditions were set as follows: feed flow solution-1.2 mL min^−1^, inlet temperature-150 ± 1 °C, outlet temperature-90 ± 2 °C, nozzle diameter tip-150 μm, atomization air flow-831 L h^−1^, and aspiration-100%.

Obtained powders were marked as follows: CMC-0.8% carboxymethyl cellulose, CMC_CLG- 0.6% carboxymethyl cellulose + 0.2% collagen, CMC_CAR-0.6% carboxymethyl cellulose + 0.2% κ-carragenan, CMC_AG-0.6% carboxymethyl cellulose + 0.2% gum arabic, CMC_GG-0.6% carboxymethyl cellulose + 0.2% hydrolyzed guar gum.

### 2.4. Physicochemical Characterization of Powders

#### 2.4.1. Determination of Dry Matter

The content of dry matter (DW) for spray-dried powders was determined according to the standard gravimetric method AOAC 930.15 [37].

#### 2.4.2. Rheological Characterization of Feed Solutions

The apparent viscosity of liquid feeds, as well as viscoelastic properties of determined non-Newtonian systems, were performed using Modular Compact Rheometer MCR 102 (Anton Paar GmbH, Graz, Austria), equipped with an air-cooled Peltier temperature control system (accuracy: 0.01 °C).

Viscosity profiles obtained upon defined shear rate range (0.1–100 s^−1^) were determined using cone (1°)-plate (diameter: 50 mm, CP50-1) measuring system, with a gap of 0.102 mm.

To define linear viscoelastic range (LVE) and determine the storage modulus G′ and loss modulus G″, amplitude sweep test was performed at 25 °C using oscillatory shear in a strain control mode at 6.28 rad s^−1^ angular frequency. For that purpose, parallel-plate (diameter: 50 mm, PP50) measuring system was employed. The range of shear strain was of 0.1–100% with the gap set to 0.500 mm. Obtained results were analyzed using rheometer software (RheoCompass^TM^, Anton Paar, Graz, Austria).

#### 2.4.3. Process Yield, Encapsulation Efficiency, and Loading Capacity

The process yield (PY, %) was gravimetrically determined by the ratio between the total recovered mass powder (m_p_) and the total dry matter in the liquid feed solution, which includes dry matter of extract and dry matter of polymer(s) (m_dw of LFS_), as stated in Equation (1):

(1) $$PY\left(\%\right)=\frac{m_{p}}{m_{\mathrm{dw}\;\mathrm{of}\;\mathrm{LFS}}}\times 100$$

The encapsulation efficiency (EE) and loading capacity (LC) of HC and individual PGs was determined by the following Equations (2) and (3), respectively:

(2) $$\mathrm{IU}\left(\%\right)=\frac{W_{i}}{W_{u}}\times 100$$

where *W_i_* represents the content of determined HC or individual PGs on dry matter of microparticles, and *W_u_* the content of determined HC or individual PGs in the initial liquid feed solution.

(3) $$\mathrm{LC}\left(\mathrm{mg}/g_{\mathrm{dw}}\right)=\frac{c\;\times V}{m_{i}}$$

where c represents the calculated concentration of analyzed HC or PGs using calibration curve, V represents the volume of dissolved microparticles, and m_i_ the dry mass of total dissolved microparticles. Finally, the results were expressed in %.

#### 2.4.4. Mean Particle Size, Zeta Potential, and Polydispersity Index (PI)

Zeta potential of analyzed powders was determined by Zetasizer Ultra (Malvern Panalytical Ltd., Malvern, UK) in Multi-Angle Dynamic Light Scattering (MADLS) mode. For the particle size measurement, all samples (1 mg mL^−1^) were diluted 100 times in methanol prior the analysis. Analysis of zeta potential and PI was conducted on undiluted samples.

#### 2.4.5. Wettability

Contact angle (CA) measurements were carried out by sessile drop method using goniometer DataPhysics OCA 20 (DataPhysics Instruments, Filderstadt, Germany), equipped with a SCA20 software (version 4.5.14). The powders (0.4–0.5 g) were pressed in compact discs to obtain smooth and flat surface. Distilled water was used as liquid and the volume of the liquid drop was 0.002 mL. The contact angle was measured immediately after the drop of liquid was deposited on the sample surface (60–80 ms).

#### 2.4.6. SEM Analysis of Powders

The morphology of the analyzed powders was determined by scanning electron microscopy (SEM) on a TESCAN Mira3 microscope (Brno, Czech Republic). Samples were sputtered with a gold layer prior to microscopic analysis to ensure electrical conductivity. Scanning was performed under a voltage of 3.8 kV and with ×800 magnification.

#### 2.4.7. Attenuated Total Reflectance-Fourier Transform Infrared (ATR-FTIR)

Structural changes in the molecular binding of pure polymers and the encapsulated delivery systems were determined by Attenuated Total Reflectance-Fourier Transform infrared spectroscopy (ATR-FTIR) analysis using Nicolet iS10 (Thermo Scientific, Waltham, MA, USA). A total of 32 cumulative scans were taken under attenuated total reflection mode in the frequency range of 400–4000 cm^−1^, with a resolution of 0.16 cm^−1^ to obtain absorption spectra.

### 2.5. In Vitro Digestion of Prepared Powders

In vitro static digestion of powders was performed according to INFOGEST 2.0. protocol [38]. Powders (0.2 g) were exposed to the simulation of gastric and intestinal phase conditions by using simulated gastric fluid-SGF (pH = 3), simulated intestinal fluid-SIF (pH = 7), and gastric enzymes. All electrolyte solutions were prepared according to the protocol.

Firstly, pepsin was weighted (New Classic ML204/01, Mettler Toledo, Zurich, Switzerland) and dissolved into the SGF electrolyte buffer solution (enzyme activity-2000 U mL^−1^ on the total volume of buffer used). After 120 min of gastric phase, gastric chyme was mixed (1:1, *v*/*v*) with previously prepared mixture of pancreatin (1 mg mL^−1^) and bile salts (2.5 mg mL^−1^) dissolved in SIF electrolyte buffer solution. Temperature (37 °C) and homogeneous mixing on magnetic stirrer (SMHS-6, Witeg Labortehnik GmbH, Wertheim, Germany) were kept constant. Simultaneously, blank sample (buffer solutions + enzymes) was also tested. HC release content was spectrophotometrically evaluated after each sampling in the interval of 5-180 min [36]. Results were expressed in mg of echinacoside equivalents per gram of dry matter of jelly sample (mg eq ech g^−1^ dw).

### 2.6. Formulation of Starch-Based Jelly Rolls

Five candy formulations with different combinations of starches were developed (Table 1).

First, sweetener slurry was prepared by mixing 75% sucrose and 25% glucose syrup in distilled water on an electric hotplate (Iskra, Ljubljana, Slovenia) and cooked until 125 ± 0.1 °C was achieved. During the heating, the temperature was precisely controlled with a digital thermometer (Testo 108-2, Titisee-Neustadt, Germany). Simultaneously, mixture of starch and water was heated, until forming a gel-like structure. Then, cooked sugar slurry was added in servings to previously gelatinized kudzu starch granules (1:5 ratio, water:starch, *w*/*w*) or their combination with constant mixing. Mixture of slurry and hydrocolloid(s) was heated and constantly mixed on electric plate. At the end of cooking, dissolved mixture of citric acid, liquid raspberry aroma, liquid red color, and milled freeze-dried raspberries (electric mill United Favor Development, Hong Kong, China) were added.

Chocolate-based filling was prepared by adding 2.2% of encapsulated extract (calculated on the total weight of the filling) into the previously prepared oil–chocolate mixture (shea butter:coconut oil, 1:4, *w*/*w*, mixed with pre-melted dark chocolate in 1:4 ratio, *w*/*w*).

Finally, the sweetener starch blend was applied in a thin layer (app. 5 mm) to the starch-coated film PP and then filled with the enriched chocolate filling. The aim was to apply 30% of the filling to the jelly shell. After that, the candy was rolled up in the form of a roll-shaped cake and wrapped in a transparent film coated with starch to keep its shape.

### 2.7. Determination of Amylose/Amylopectin Content in Starches

The commercial amylose/amylopectin enzymatic kit was used for the spectrophotometric determination of amylose and amylopectin content in corn and kudzu starches (Megazyme Ireland International, Ltd., Bray, Ireland). Procedure was followed by manufacturer instructions. The determined results of amylose and amylopectin values were, as follows: 18.50 ± 0.30% and 81.50 ± 0.26% for kudzu starch, and 21.48 ± 0.27% and 78.52 ± 0.23% for commercial corn starch, respectively.

### 2.8. Determination of Dry Matter in the Jelly Rolls

The content of dry matter (DW) for jelly rolls was determined according to the standard gravimetric method AOAC 930.15 [37].

### 2.9. Bioactive Evaluation of Formulated Jelly Rolls

#### 2.9.1. Extraction of PGs and Methylxanthines

Firstly, defatting procedure of all jelly roll samples was performed according to Adamson et al. (1999) [39]. Then, methylxanthines and PGs were extracted from the defatted jelly solid with 5 mL of 70% methanol (*v*/*v*). The extraction (30 min, 65 °C) was performed in an ultrasonic bath (S60H Elmasonic, Elma Schmidbauer, Singen, Germany), followed by additional homogenization on a magnetic stirrer (SMHS-6, Witeg Labortehnik GmbH, Wertheim, Germany) (15 min, 400 rpm) and centrifugation (5000 rpm, 10 min, 4 °C) (SL 8R, Thermo Scientific, Waltham, MA, USA).

The extraction and centrifugation procedures for each sample were carried out three times in total, and the supernatants of each sample were combined in a 10 mL volumetric flask and filled with 70% (*v*/*v*) methanol up to the mark.

#### 2.9.2. HPLC-UV-DAD Analysis

The quantification of PGs in jelly rolls was analyzed by HPLC-UV-DAD, applying the same chromatographic conditions as stated in the previous study of Mandura Jarić et al. (2023) [30].

Results were expressed in mg equivalents of echinacoside per gram of dry matter of samples (mg eq ech g^−1^ dw of jelly).

### 2.10. Texture Analysis

Texture profile analysis (TPA) was performed on a texture-measuring device (Texture Analyzer, TA.HD.plus, Stable Micro System, Godalming, UK) coupled with Texture Exponent software 4.0 for data processing. Samples were horizontally centered under the flat cylindrical probe (5000 g). Setting parameters were as follows: test speed-1 mm s^−1^, compression speed-5 mm s^−1^, return speed after compression-5 mm s^−1^, compression depth-1 mm, number of compression cycles-2, and time interval between two compressions cycles-5 s. The monitored parameters are hardness, springiness, cohesiveness, gumminess, and chewiness. Measurements were obtained at room temperature.

### 2.11. Sensory Analysis

Formulated starch-based jellies were sensory evaluated at the Faculty of Food Technology and Biotechnology (University of Zagreb, Zagreb, Croatia) following ISO Standard No. 11035 with some modifications [40,41]. Sensory panel formed of 20 educated panelists (aged 25 to 50) with previous experience in the assessment of similar confectionery products was selected. Training sessions for the panelists were conducted for identification and quantification of descriptive attributes. Jelly rolls without filling were also served for better insight into specific textural profiles as the result of used starch type. Intensity of selected sensory attributes, i.e., hardness, adhesiveness, chewiness, consistency, sweetness, bitterness, and aftertaste, was assessed for starch-based jellies with incorporated encapsulated extract, on a scale from 9 (“extremely intense”) to 1 (“not perceived”) by applying descriptive sensory analysis. Also, evaluation of overall acceptance was conducted by hedonic-point scale, with “9” representing “extremely desired quality”, and “1” “extremely disliked quality”. Bitterness and aftertaste were also scored for filled jelly rolls without incorporated extract.

### 2.12. In Vitro Digestion of Jelly Roll

Jelly formulation evaluated with the highest score in overall acceptance was introduced to in vitro simulation of digestion. In vitro digestion of selected jelly was performed according to INFOGEST 2.0. protocol [38], as stated in Section 2.5, with some additions.

Oral phase of selected jelly roll formulation (one serving-6g) was conducted in simulated salivary fluid (SSF) mixture (pH = 7), with previously dissolved human salivary α-amylase (final enzyme activity in mixture = 75 U mL^−1^). After 2 min, oral bolus was mixed with prepared SGF-enzyme mixture (1:1, *v*/*v*), and exposed to the gastric phase (pH = 3, 120 min). Finally, intestinal phase was also conducted by mixing the gastric chyme with SIF-enzyme mixture (pH = 7).

Simultaneously, blank sample (simulated buffer solutions + enzymes + jelly without incorporated extract in the filling) was taken simultaneously in the same time intervals. To compare the kinetic release of encapsulated extract, jelly sample with the non-encapsulated, lyophilized extract was also introduced to the same in vitro digestion conditions.

The subtracted absorbance of tested jelly sample (with encapsulated and non-encapsulated extract) and the absorbance of blank sample was used for the calculation of the concentration of HC, released in each time interval, and expressed as mg of equivalents of echinacoside per one gram of dry matter of jelly sample (mg eq ech g^−1^ of dw).

### 2.13. Statistical Analysis

One way ANOVA with Tukey post-hoc test (significance level, α < 0.05), and *t*-test were conducted on every set of results in Statistica 13.3 software package (TIBCO Software Inc., Palo Alto, CA, USA). FTIR graphs were acquired in OriginPro 2023b (10.5) (OriginLab Corporation, Northampton, MA, USA). GraphPad Prism 10.1.2 (trial version; GraphPad Software, Boston, MA, USA) was employed for graphical display of rheological data.

## 3. Results and Discussion

### 3.1. Determination of HC and Phenolic Compounds in Extract

To this end, mostly phenolic acids and flavonoids were quantified in *T. montanum* extracts [8,9,10]. However, it can be seen that *T. montanum* extract, as a generally understudied plant species in terms of bioactive profile, is shown to be a notable source of PGs, in comparison to its commercial plant sources (Table 2).

For example, the reported contents of echinacoside and verbascoside in *C. salsa* (10.98 mg g^−1^ and 9.44 mg g^−1^, respectively) [42], and poliumoside in *C. sinensis* (3.4–36.2 mg g^−1^) [43] are similar to the ones obtained for *T. montanum* extract (echinacoside: 23.54 mg g^−1^ dw, verbascoside: 7.90 mg g^−1^ dw, poliumoside: 21.72 mg g^−1^ dw). In comparison to the study of Esposito et al. (2020) [44] on *Ajuga reptans* L. extract as a well-known source of teupolioside (2.8% on dry weight of extract), *T. montanum* seems to be a potentially valuable source as well (6.93 mg g^−1^ dw). Taking into account the quantified PGs in this study by HPLC analysis, it can be seen that these compounds mostly contribute (~72%) to the evaluated content of HC in *T. montanum* extract.

### 3.2. Rheological Characterization of Feed Solutions

**Figure 1:**
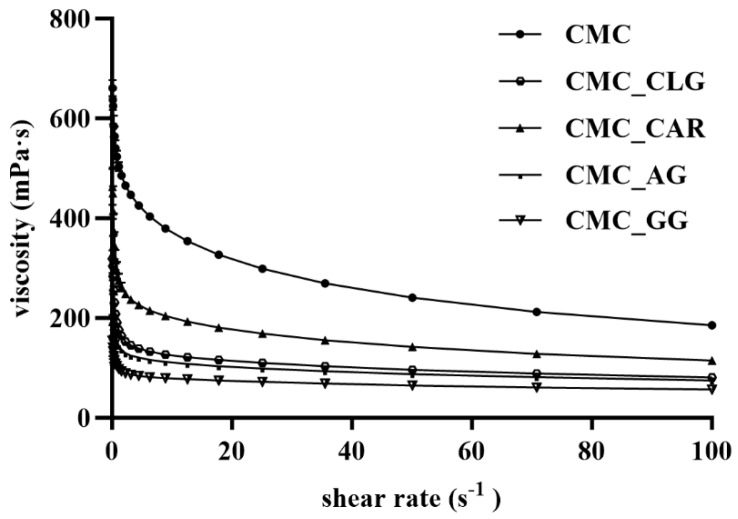
Viscosity curves of tested feed solutions within the shear rate range of 0.1–100 s^−1^ (CMC-carboxymethyl cellulose, CMC_CLG-carboxymethyl cellulose+collagen, CMC_CAR-carboxymethyl cellulose+κ-carrageenan, CMC_AG-carboxymethyl cellulose+gum arabic, CMC_GG-carboxymethyl cellulose+guar gum).

Due to the large influence on droplet size formation, process yield, and consequently encapsulation efficiency, the viscosity of the prepared feed solutions was measured. Figure 1 shows viscosity curves of feed solutions within the shear rate range of 0.1–100 s^−1^.

The results showed a shear-thinning behavior for all tested samples, as the apparent viscosity decreased with increasing shear rate between 0.1 and 20 s^−1^. The more the shear rate increases, the more the polymer network deteriorates, causing the viscosity to decrease until it reaches a constant value at higher shear rates (20–100 s^−1^). This is due to the anisotropic property of polymer materials, i.e., the disentanglement of the polymer chains by breaking the intermolecular bonds (van der Waals and hydrogen bonds) and the alignment of the molecules along the applied shear force [45,46]. Regarding the influence of the carrier material, 0.2% (*w*/*w*) substitution of CMC with gum arabic, guar gum, hydrolyzed collagen, and κ-carrageenan significantly decreased the viscosity of the pure CMC solution (298.76 mPa·s at 25 s^−1^) by 43–76%, depending on the biopolymer used. The CMC_AG and CMC_GG feed solutions had the lowest apparent viscosity of 98.97 mPa·s and 72.08 mPa·s, respectively, at 25 s^−1^. This is likely the result of hydrolysis pretreatment of the guar gum and collagen used, as well as the polyelectrolyte nature of CMC in the presence of other solutions with similar character, e.g., GA, CAR, or CLG, which provide charge shielding in between. In addition, branched structures such as those of GA or CAR lead to compact molecules with a lower hydrodynamic volume in the solution, so that it can only become viscous at high concentrations. In contrast, the high viscosity of the CMC solution is the result of linearly aligned chains that provide intermolecular entanglements between the molecules at much lower concentrations [47,48]. It is known that the molecular weight of the polymers, the concentration of the solution, and the nature of the interaction between polysaccharides and/or proteins influence the final viscosity [49]. 

Given the significant influence of the viscoelastic properties of the feed solutions on atomization and process yield, storage modulus (G′) and loss modulus (G″) were determined by the amplitude sweep test, as indicators of elastic and viscous behavior, respectively (Table 3).

**Figure 2:**
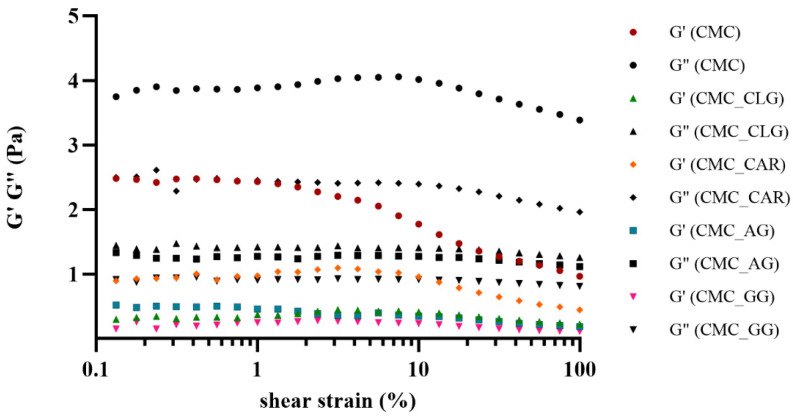
Storage (G′) and loss (G″) moduli as a function of shear strain for tested feed solutions. CMC-carboxymethyl cellulose, CMC_CLG-carboxymethyl cellulose+collagen, CMC_CAR-carboxymethyl cellulose+κ-carrageenan, CMC_AG-carboxymethyl cellulose+gum arabic, CMC_GG-carboxymethyl cellulose+guar gum.

Figure 2 shows two characteristic regions: the linear viscoelastic region (LVE), which is defined as the strain region in which the structure of the sample is preserved, and the nonlinear viscoelastic region, which represents the strain region in which the structure disintegrates. The LVE range for CMC was observed to be between 0.1 and 1.78%, whereas it was higher for all the other feed solutions, i.e., in the range of 0.1–10%.

The relationship G′< G″ was observed for all tested samples in the LVE range, indicating fluid behavior. Thus, the replacement of CMC (0.2%, *w*/*w*) by GG, GA, CAR, or CLG had no effect on the overall liquid state. Since G′ values in the LVE region are used for evaluating the strength of the viscoelastic material, i.e., for the intermolecular interactions, [50,51,52], it was shown that the added polymers reduced the strength of the samples, reducing the interactions between the polymer chains. Compared to the CMC sample (G′ = 2.28 Pa at 1% of shear strain), the strongest decrease in the G′ value was observed for the CMC_GG (0.32 Pa at 1% of shear strain) and CMC_CLG (0.45 Pa at 1% of shear strain) samples (*p* < 0.05). The calculated tan δ values, a numerical parameter for the ratio of lost to stored energy per deformation cycle, also confirmed a more fluid-like behavior [53,54]. 

### 3.3. Physicochemical Characterization of Powders

#### 3.3.1. Process Yield, Encapsulation Efficiency, and Loading Capacity

In terms of spray-drying efficiency and potential industrial scalability, the process yield is an important parameter to determine. In this study, CMC, used both individually and combined as wall material, was shown to be very appropriate for the production of powders with low water content (<5.52%). As exhibited in Table 4, the replacement of 0.2%, *w*/*w*, CMC, with GG, GA, CAR, or CLG significantly improved the spray-drying efficiency.

Process yield ranged from 32.55 to 55.00% following the order: CMC_KAR > CMC_CLG > CMC_GG > CMC_AG > CMC. This is in agreement with the study of Castro López et al. (2021) [55], who reported the improvement in PY of spray-dried *M. oleifera* extract by employing locust bean (57.2%) and tragacanth gum (67.8%) with CMC in comparison to the CMC alone (56.4%). Generally, PY above 50% can be considered satisfactory in terms of lab-scale production [56]. A negative correlation between the viscosity values and process yield also could be observed (r = −0.78), indicating the significant effect of proper viscoelastic properties on the drying efficiency. The product loss during the process is obviously related to the remaining particle residues at the cyclone entrance and outlet filter, as well as the surface phenomenon called stickiness, causing the deposition of powdery material on the dryer chamber wall due to the interaction between the cohesion forces between the particles and adhesive forces, mainly liquid bridging forces [57]. It could be assumed that the higher viscosity of the feed solution affected the lower evaporation rate, thus promoting the particle agglomeration at the spraying cylinder. Apart from viscoelastic properties, process yield is affected by the other feed material properties, i.e., sticky-point temperature and/or glass transition temperature, hygroscopic properties, and processing parameters, i.e., spray gas flow, spray cone angle, inlet/outlet temperature, etc. [58,59,60].

Regarding the efficiency of the spray-drying technique and polymer combinations in the retention of HC derivatives from *T. montanum* extract, results showed relatively high encapsulation efficiency (EE) of HC in a range of 68.67–97.31% (Table 5).

Generally, the employment of combined polymers improved the EE for both, HC derivatives and PGs, in comparison to the individually applied CMC. On the contrary, Castro López et al. (2021) [55] found that the spray-drying application of carboxymethyl cellulose alone was more successful in achieving higher EE for TPC and TFC (total flavonoid content) from *Moringa oleifera* extract than combining it with selected polysaccharides. Although all combinations in this study seem to be relatively appropriate for the PGs delivery, CMC_AG and CMC_GG were distinguished in the matter of achieving almost 100% EE for examined phenolic compounds, i.e., 103.31 and 101.66% for teupolioside, respectively, and between 90.61 and 95.79% EE for echinacoside, stachysoside A, poliumoside, and verbascoside. Despite the great lack of studies exploring the potential of spray-drying preparation of PGs-based delivery systems, Leyva-Jiménez et al. (2020) [61] reported the promising potential of spray drying in the preparation of inulin and maltodextrin powders enriched with lemon verbena green extract, analyzed in EE > 71.22% for total PGs, among which verbascoside was the most dominant. It is worth mentioning that studies imply the importance of various parameters of wall material and phenolic agents, e.g., chemical complexity, molecular weight of polymer and side chains, branching ratios, solubility of polyphenols, different types of functional groups, and their conformation on the interaction character between polyphenols and selected carriers [62,63].

With respect to the loading capacity, the amount of HC loaded on the macroparticles was in the range of 20.15–23.12%. As expected, echinacoside was the dominant PG in all analyzed microparticles (4.2–6.13%), accounting for 20–30% of measured HC.

#### 3.3.2. SEM Analysis of Spray-Dried Powders

**Figure 3:**
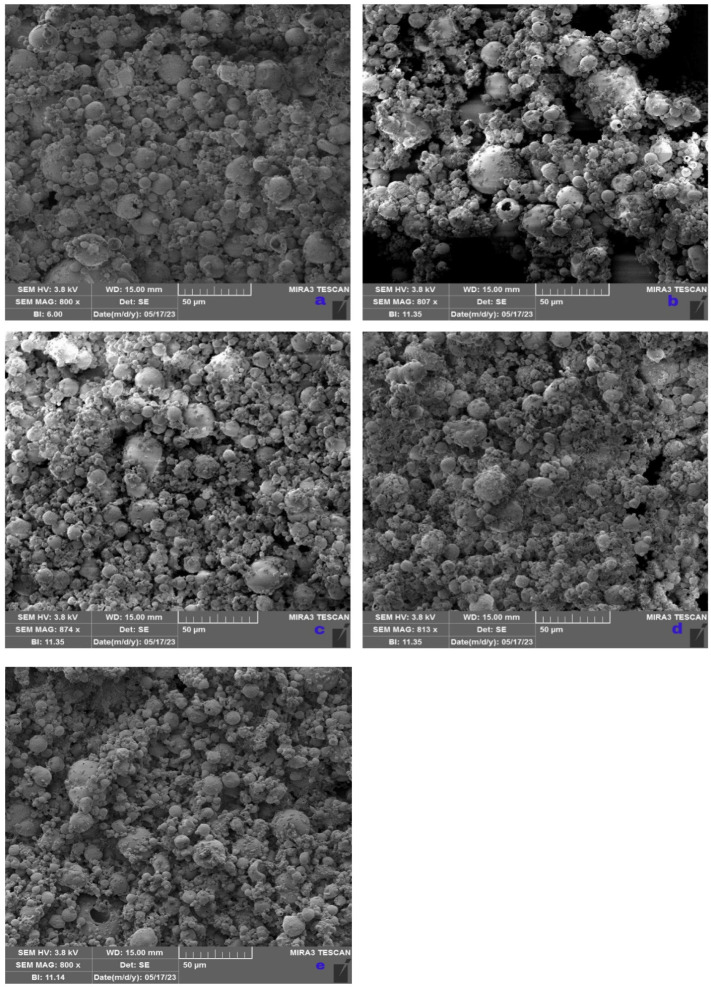
SEM images of microparticles (magnification 800×) (**a**) CMC, (**b**) CMC_CLG, (**c**) CMC_CAR, (**d**) CMC_AG, (**e**) CMC_GG.

The surface morphology of the spray-dried powders was evaluated by SEM analysis (Figure 3a–e). It can be seen that spray-dried microparticles of all examined samples generally exhibited agglomerated and mostly spherical shapes of various sizes.

The particles within CMC, CMC_CLG, and CMC_GG also showed the presence of hollows, fissures, and collapses, with some semi-spherical and irregular shapes (Figure 3a,b,e). Similar results were obtained for CMC microparticles in the study of Castro López et al. (2021) [55], showing a micrograph of agglomerate structures with surface collapses. The correlation between high values for PI and the visualization of agglomerate structures via SEM is evident. The agglomeration phenomenon as the consequence of short physicochemical interaction between collided particles is commonly related to the spray-drying process, and it may have a positive impact on the stability and release of targeted compounds due to the outer particle layer, leading to the protection of inner particles in conglomerates [64,65]. Also, this study confirms the impact of GG and hydrolyzed CLG substitution for CMC (25%, *w*/*w*) on shape transformation causing irregularities, as stated in other studies [66,67], although concave shapes were not detected in this study. However, microparticles obtained with the addition of CAR and AG presented a round-like shape with a non-collapsed and smooth surface (Figure 3c,d). In this case, AG addition (25%, *w*/*w*) did not have a negative influence on forming concavities and quasi-spherical forms with rough surfaces, as the employment of 100% AG [68]. A smooth surface and the absence of pores are desirable qualities that may greatly influence oxidation prevention and reduced interaction with other particles [69].

#### 3.3.3. Zeta Potential, Polydispersity Index (PI), and Mean Particle Size

To evaluate the possible electrostatic interaction between microparticles in the methanol solution (pH = 6), and their uniformity in terms of particle size, zeta potential and polydispersity index were measured, respectively (Table 4). As can be seen, zeta potential was mostly affected by the addition of polysaccharides or proteins instead of CMC as the main wall material (*p* < 0.05). Moderate negative zeta potential of all CMC samples (−17.91–−11.76 mV) is the result of deprotonated carboxyl groups of the main used WM. The substitution of CMC with AG caused a significant additional decrease (CMC_AG: −17.91 mV) whereas CMC_GG exhibited an increase in zeta potential (−11.76 mV), probably due to the presence of deprotonated hydroxyl and carboxyl groups in AG and uncharged character of GG, respectively. Observed results indicate the relatively low stability in aqueous systems. These results are related to the high PI values for all spray-dried powders (0.82–1.00), implying high heterogeneity of particle size within each sample, and thus, a high potential for microparticle agglomeration due to the lack of electrostatic repulsion [70].

In terms of the results for mean particle size (µm), the employment of non-standard nozzle tip diameter (150 µm) resulted in the formation of fine microparticles in the range of 2.39–3.09 µm for all analyzed CMC-based powders. Major significant differences in the evaluated parameters were not detected when employing the additional polymer instead of CMC. Some larger deviations in the mean particle sizes can be explained by the wide spread of particle size distribution, confirmed by the PI > 0.82.

#### 3.3.4. Wettability

Contact angle measurements (θ°, i.e., CA) were employed for the qualitative evaluation of the hydrophobic/hydrophilic character of the powder surface. As can be seen in Table 4, all samples showed low wettability due to the CA values measured over 90°, whereby CMC (θ: 144°), CMC_AG (θ: 140°), and CMC_GG (θ: 136°) blends showed the most pronounced hydrophobic nature. Generally, CMC could be considered a hydrophobic derivative from cellulose, which could be used for the improvement of the hydrophobic character and barrier properties of biodegradable films [71,72]. Also, the roughness of the powder surface could influence some higher values of contact angles [73]. On the other side, the addition of CLG and CAR significantly decreased the CA (*p* < 0.05), thus improving the wettability of delivery systems. This is probably due to the amphiphilic nature of CLG, as well as the excess of CAR sulfate groups, influencing higher hydrophilicity, i.e., the capability to interact with water.

#### 3.3.5. ATR-FTIR

**Figure 4:**
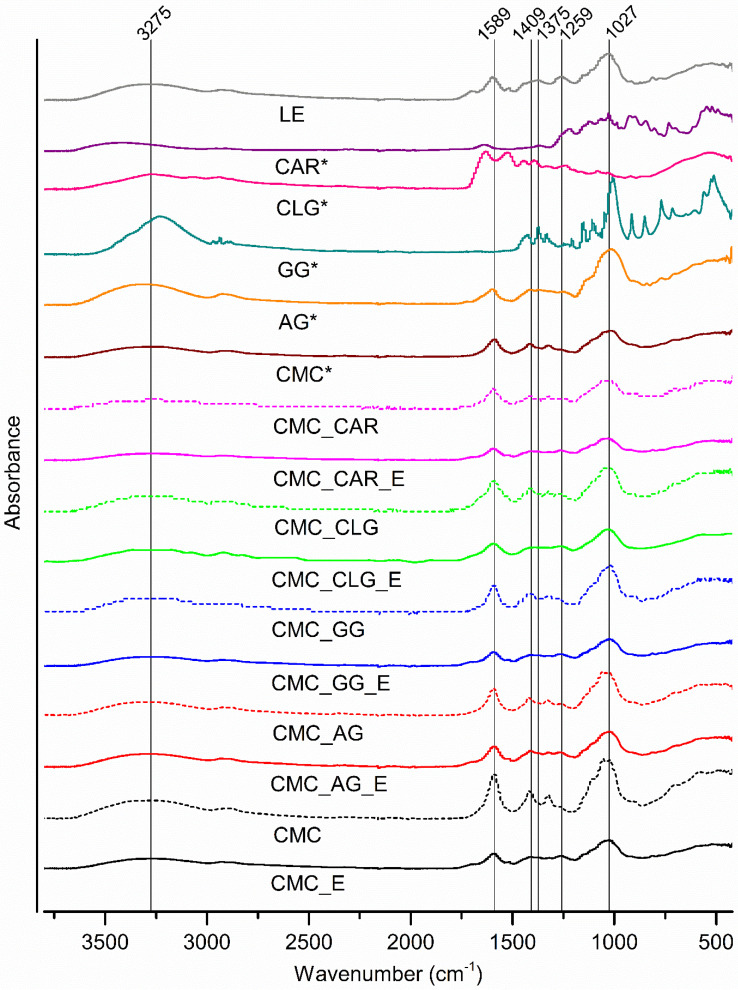
FTIR spectra of CMC-based microparticles. CMC_E-carboxymethyl cellulose+extract, CMC_CLG_E-carboxymethyl cellulose+collagen+extract, CMC_CAR_E-carboxymethyl cellulose+κ-carrageenan+extract, CMC_AG_E-carboxymethyl cellulose+gum arabic+extract, CMC_GG_E-carboxymethyl cellulose+guar gum+extract; CMC—spray-dried carboxymethyl cellulose, CMC_CLG-spray-dried carboxymethyl cellulose+collagen, CMC_CAR-spray-dried carboxymethyl cellulose+κ-carrageenan, CMC_AG-spray-dried carboxymethyl cellulose+gum arabic, CMC_GG-spray-dried carboxymethyl cellulose+guar gum; CMC*-untreated carboxymethyl cellulose, AG*-untreated gum arabic, GG*-untreated guar gum, CLG*-untreated hydrolyzed collagen, CAR*-untreated κ-carragenan, LE-lyophilized extract.

To determine the possible interaction between functional groups of used polymer carriers and polyphenols in *T. montanum* extract, ATR-FTIR analysis was conducted (Figure 4).

As can be observed, the characteristic bands for *T. montanum* extract were in a broad range of 900–1800 cm^−1^. The multiple overlapping bands, with the most pronounced peak at 1027 cm^−1^, arising mostly from C-O-C and C-O symmetric stretching, confirm the presence of glycosidic bonds between hydroxytyrosol and sugar moieties in the PGs. The broad band at ~3500 cm^−1^ confirms the presence of hydroxyl groups. The signals at 1259 cm^−1^ (C-O), 1375 cm^−1^ (C-H; O-H), 1589 cm^−1^ (C-C; C=C), 2930 cm^−1^ (C-H), and 3275 cm^−1^ (O-H) wavenumbers are related to the aromatic structures of polyphenolic compounds.

Along with encapsulated CMC-based delivery systems, FTIR spectra were recorded for all pure polymers, as well as for all spray-dried, spray-dried polymer combinations without the extract.

The functional groups of pure CMC are corresponding to those in the literature [55,74]. The appearance of broad peaks at 3250, 1589, 1413, and 1324 cm^−1^ are attributed to the presence of asymmetric and symmetric OH stretch within H-bonding or OH stretch from carboxyl groups. The peak at 1019 cm^−1^ represents bending modes in the cellulose backbone from carbon–oxygen stretching (C-O-C). In the spectrum of AG, bands of relatively high intensity can be observed at 3311, 2926, 1597, and 1015 cm^−1^, which correspond to O-H stretching, C-H stretching vibration, COO^- -^ symmetric stretching, C-O-C- glycosidic bonding between sugar moieties, and O-H vibration band stretching in polysaccharide chains, respectively [75,76]. Regarding the FTIR spectrum of GG, the broad bands were detected at 3232 cm^−1^, which is aligned with the stretching of free, hydrogen-bonded or -attached hydroxyl groups to carboxyl groups, along with the peaks at 2937 cm^−1^, 1425 cm^−1^, 1373 cm^−1^, and 1331 cm^−1^, which are predominantly related to the C-H stretching within a galactomannan polymer backbone. The sharp and intensive peaks in the range between 400 and 1200 cm^−1^ (1152 cm^−1^, 1009 cm^−1^, 914 cm^−1^, 851 cm^−1^, 769 cm^−1^, 511 cm^−1^) correspond to the stretching vibrations of β(1→4) and α(1→6) glycosidic bonds between mannose and galactose units, as well as OH-bending within primary alcoholic and carboxyl groups [77,78]. The characteristic absorption bands were observed for CLG, i.e., amide I at 1630 cm^−1^ (dominant C=O stretching within peptide bonds along the backbone), amide II at 1524 cm^−1^ (primarily in-plane N-H bending of primary amines in peptides, along with C-N and C-C stretching) and amide III at 1240 cm^−1^ (dominant C-N stretching of secondary amides). Also, amide A (3270 cm^−1^) and amide B (2941 cm^−1^) are detected, representing the asymmetric stretching of C-H_2_, O-H, and N-H_2_ functional groups, respectively [79]. For the CAR, the broad band (3100–3700 cm^−1^) with the maximum peak at 3408 cm^−1^ corresponded to the O-H stretching, whereas the broad band between 1210 and 1260 cm^−1^ (peak at 1219 cm^−1^) indicates the presence of sulphate esters [80]. The specific sharp peaks at 923 cm^−1^ and 846 cm^−1^ are assigned to the 3,6-anhydro-galactose residue and D-galactose-4-sulphate, respectively. In addition, intensive bands between 1148 and 1028 cm^-−1^, and 800 and 700 cm^−1^, are presumably attributed to the stretching and bending in pyranose units within polysaccharide chains [81,82].

All CMC-based delivery systems without encapsulated extract exhibited very similar absorption bands and peaks as the CMC itself (CMC*), which was due to its dominant presence in all tested combinations (75%, *w*/*w*). As can be observed, relative peak shifts compared to the CMC* at 3250 cm^−1^, 1046 cm^−1^, and 1019 cm^−1^, were detected in all spray-dried combinations as follows: CMC- 3260 cm^−1^, 1054 cm^−1^ and 1023 cm^−1^, CMC_AG- 3287 cm^−1^, 1054 cm^−1^ and 1027 cm^−1^, CMC_GG- 3264 cm^−1^, 1022 cm^−1^, CMC_CLG- 1326 cm^−1^, 1022 cm^−1^, CMC_CAR- 3247 cm^−1^, and 1050 cm^−1^ and 1023 cm^−1^. Such changes are presumably related to the introduction of the secondary polymers, modifying intermolecular interactions, e.g., H- bonding between hydroxyl or carboxyl groups of polysaccharide chains, and amino groups within hydrolyzed collagen, as well as the stretching intensity of dominant glycosidic bonds.

FTIR spectra of enriched CMC-based powders did not show any new peaks. However, a relative decrease in the intensity of absorption band peaks around 3275 cm^−1^, 1589 cm^−1^, and in the “sugar footprint” area, i.e., 1409 cm^−1^, 1323 cm^−1^, and 1027 cm^−1^ was observed, indicating interactions between polymer carriers and extract components, presumably through hydrogen bond formation [83]. So far, many studies revealed that the non-covalent interaction between polyphenols and polysaccharides or proteins was predominantly driven by hydrogen bonds, along with hydrophobic interactions, electrostatic interactions, and van der Waals forces [84,85].

### 3.4. In Vitro Digestion of Microparticles

**Figure 5:**
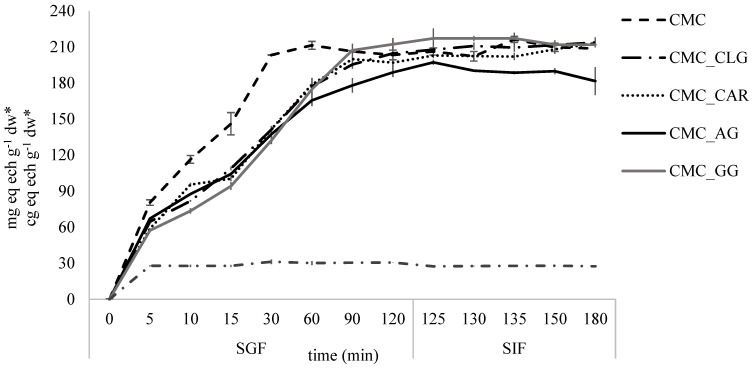
In vitro digestion of CMC-based microparticles. mg eq ech g^−1^ dw*—expressed on dry matter of microparticles; cg eq ech g^−1^ dw*—expressed on dry matter of lyophilized extract.

To evaluate the release profile of total PGs as caffeic acid derivatives from CMC-based delivery systems and their bioaccessibility in the simulated gastrointestinal conditions, the INFOGEST static in vitro digestion method was employed. Figure 5 shows the quantified total HC derivatives from the non-encapsulated extract and spray-dried encapsulated extract analysis during the gastric phase (120 min) and intestinal phase (60 min).

It can be seen that CMC showed the fastest release of HC (~38%) in the first 5 min of the gastric phase, whereas samples CMC_CAR and CMC_AG exhibited the best retention of HC at the beginning of the simulated gastric process (~73%). A similar trend continued during the rest of 115 min, with CMC_GG and CMC_CLG showing the satisfactory release kinetic of HC. After 120 min of gastric conditions, results showed that approximately 95–100% of encapsulated HC from the extract was released from all analyzed delivery systems, except for CMC_CAR (~92%). In comparison to spray-dried encapsulated extract, lyophilized extract at the beginning of the digestion process showed ~100% release of measured HC. Analogous to the results in this study, Wei et al. (2021) [86] also reported the stability of caffeic acid derivate, i.e., caffeic acid phenethyl ester, in the intestinal alkaline environment.

On the other side, the potential of PGs cleavage into glycone part and free phenolic forms, e.g., caffeic acid, as the result of hydrolysis and enzymolysis under gastrointestinal conditions also must be taken into account when interpreting the release kinetics of caffeic acid derivatives.

However, decomposition may not happen for all PGs due to their large structure versatility (e.g., number of sugar moieties, number of ester and ether bonds, types of glycosidic bonds, etc.) [87]. Furthermore, the unchanged amount of HC released at the end of the intestinal phase argues for the relative preservation of PGs under the conditions tested, as many studies have reported the oxidation of free caffeic acid in the intestinal alkaline environment [88,89,90].

It can be concluded that high PI values and low electrostatic repulsions between microparticles as consequences of agglomeration structures, as well as low wettability of powders probably contributed to the slower release of HC content. All the tested combinations of two biopolymers showed great potential for progressive delivery of caffeic acid derivatives, compared to the use of CMC alone.

### 3.5. Characterization of Formulated Jelly Rolls

#### 3.5.1. Bioactive Potential

**Figure 6:**
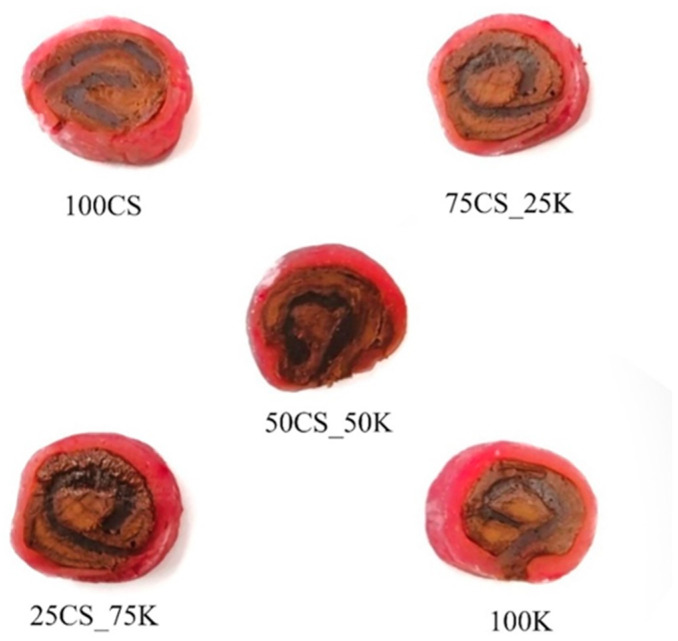
The appearance of the formulated jelly rolls.

To determine the total content of PGs and methylxanthines per serving of formulated jelly rolls (Figure 6), HPLC analysis was conducted.

From Table 6, it can be seen that approximately 8–16 mg of the total analyzed BC content, including echinacoside, teupoliuoside, stachysoside A, poliumoside, verbascoside, theobromine, and caffeine, can be introduced per one serving (6 g).

Regarding PGs, these compounds accounted for approximately 58% of total BC content. One potential drawback of food fortification with BC, especially polyphenols, is the actual amount that could be incorporated in the final product to achieve notable bioactive enrichment while relatively preserving the desirable texture and sensory attributes of the food product. To this end, many studies successfully implemented various bioactive sources in form of liquor extract or encapsulated delivery systems into gummy or jelly candies, such as sage byproduct extract [91], rosemary extract [92], hibiscus extract-based microcapsules [93], and double drum-dried Lamduan extract [94], resulting in the food model enrichment with 30–38 mg GAE 100 g^−1^ (TPC), 197–411 µg GAE g^−1^ (TPC), 1.99 mg GAE 100 g^−1^ (TPC), and 1.4 mg cyanidine-3-glucoside E g^−1^ dw (total anthocyanin content), respectively. When comparing the bioactive potential of the final product with the previous studies, a minimum 10 times higher fortification was achieved in this study, only by measuring individual PGs content by HPLC.

#### 3.5.2. Texture Profile Analysis (TPA)

The instrumental analysis of the texture profile was conducted in order to predict the desired attributes of the food product as well as to correlate the results with the sensory acceptance (Table 7).

Regarding the results of TPA, it could be seen that instrumentally measured cohesiveness, springiness, chewiness, resilience, and adhesiveness were unchanged (*p* > 0.05), independently of applied combinations of corn and kudzu starch. In terms of hardness evaluation, lower values for 100CS (794.94 g) in comparison to the sample 100K (978.94 g) were observed (*p* < 0.05). So far, there are no studies evaluating the differences in texture parameters of kudzu or commercial corn starch-based pastes or gels, although generally, some studies reported a positive correlation between the amylose content and hardness in formed gel systems [95,96]. When suspended in water and heated, starch granules are subjected to hydration, swelling, and further structure disintegration into paste, due to the dissolved crystalline structure of starch granules and leaching of mostly amylose in the surrounding medium. During the cooling process, the gel structure is formed through a process of retrogradation as a consequence of reassociating the previously leached amylose chains via hydrogen bonds into a crystalline structure, with dispersed and swallowed amylopectin molecules. In the initial retrogradation phase, amylose content greatly affects the gel hardness. The more leached amylose content in the paste during the gelatinization process, the more rebuilt crystalline structures, entrapping the amylopectin molecules and resulting in a more rigid structure. Since kudzu starch was characterized with less amylose fraction (18.50%) than commercial corn starch (21.48%) in this study (*p* < 0.05), no positive relation between the hardness of formulated jelly rolls and amylose content can be observed in between. The reason is probably insufficient sensitivity of the instrument toward a relatively small yet numerically significant difference between quantified amylose in analyzed starches.

#### 3.5.3. Sensory Evaluation

**Figure 7:**
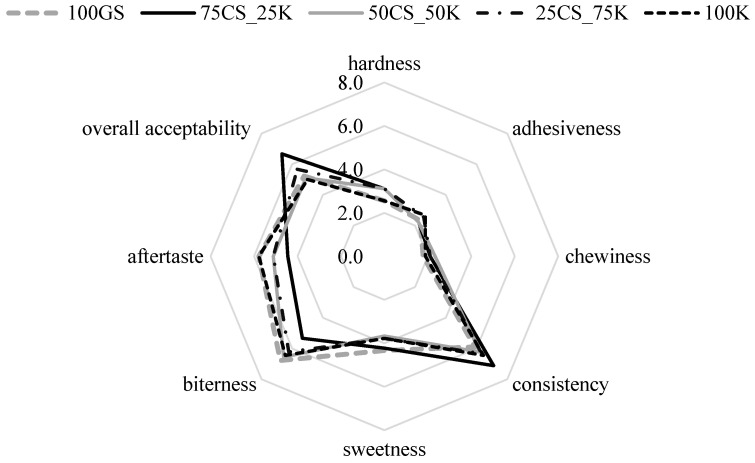
Sensory analysis of formulated starch-based jelly rolls.

Although texture analysis provides a useful screening of diverse and mutually correlated sensory attributes for a given food product, sensory evaluation is still an unavoidable step to gaining insights that meet real consumer expectations. Considering the authentic combination of chocolate filling and smooth starch shell, hardness, chewiness, adhesiveness, and consistency were defined as the most relevant for the evaluation on the hedonistic intensity scale from 1–9 (Figure 7).

Adhesiveness was meant for the sticky feeling during consumption, whereas consistency was defined as the integrated sensation of creamy and smooth perception. Although some sort of sticky feeling was expected due to the presence of the starch-based jelly matrix as a natural adhesive, formulated jelly rolls were perceived as low-sticky (score: 2.2–2.7). Results showed that there was no difference between all five formulations regarding chewiness (score: 1.8–2.3) and hardness (score: 2.6–3.1), with the absence of stickiness, elasticity, and hardness during mastication. A slight difference was observed in evaluating consistency, with the 75CS_25K perceiving the softest and smoothest texture. Regarding the perception of taste attributes, bitterness, originating from the presence of methylxanthines and PGs in the filling, was the most pronounced in all samples, as expected. Taking into account slightly lower bitterness in comparison to other samples, the most desirable consistency, sample 75CS_25K scored the highest ranked overall acceptability (score: 6.7).

When relating the results of the TPA test and sensory evaluation, high positive Pearson correlation coefficients between sensory-evaluated chewiness and instrumentally determined springiness and cohesiveness could be observed (r = 0.61 and 0.76, respectively). Such results are expected, since the chewiness, generally defined as the energy required for masticating the food, is derived from the hardness, cohesiveness, and springiness. Interestingly, sensory-perceived hardness exhibited low correlations with cohesiveness (r = 0.47) and springiness (0.45), but a high correlation with sensory-evaluated chewiness (r = 0.89). Such discrepancy may be related to the low oral tactile sensitivity toward differencing the hardness and chewiness of products with distinctive soft consistency. This explanation is also supported by the strong positive correlation between the sensory-evaluated hardness and chewiness (r = 0.89).

#### 3.5.4. In Vitro Digestion of Fortified Jelly Roll

The bioaccessibility of phytochemicals from the food matrix greatly depends on the type and quantity of the present macronutrients and their interaction with targeted compounds [97]. According to the morphological properties and in vitro digestion assessment, CMC_CAR spray-dried powder was selected for the enrichment of jelly chocolate filling. Thus, the release assessment of encapsulated polyphenols from the jelly roll was also evaluated by INFOGEST simulated digestion through the oral, gastric, and intestinal phases.

**Figure 8:**
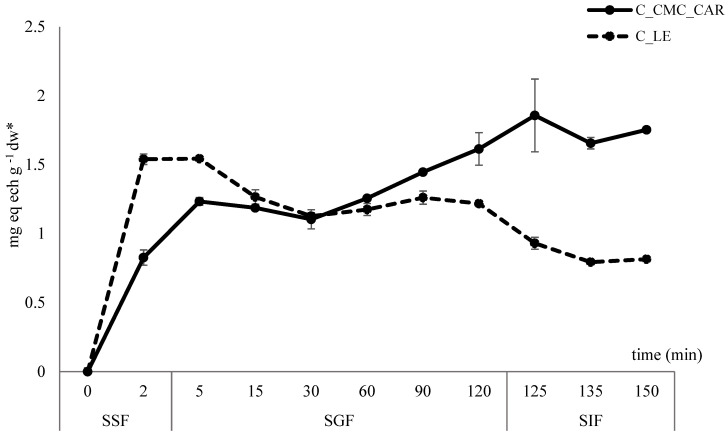
In vitro digestion of 75CS_25K jelly roll with encapsulated (C_CMC_CAR) and non-encapsulated extract (C_LE). The results for dw of jelly rolls were in the range of 90–93%; mg eq ech g ^−1^ dw*-expressed on dry matter of jelly rolls.

From Figure 8, a significant difference in the release of HC of non-encapsulated and encapsulated extract can be observed.

Interestingly, alkaline conditions and the human salivary enzyme activity in the oral bolus significantly influenced the rapid release of PGs from the filling of the control sample (with lyophilized extract), as well as the sample with encapsulated extract. Although many studies reported the minor influence of the oral phase on the bioaccessibility of, e.g., HC and/or flavonoid glycosides [98,99], especially in terms of short time exposure, some of them still observed the opposite, e.g., on kaempferol glycosides from Sanyeqing plant root [100], flavonols and flavons from *D. harra* extract [101], HC, flavans and flavons from apple peel and flesh [102], etc. Hence, the influence of the sample composition and chemical structure (e.g., presence of esther or glyosidic bonds) of targeted compounds and interactions in between is evident. Further, the degradation trend of PGs in both gastric and intestinal phases was observed for the control sample, preserving only approximately 50% of HC at the end of simulated digestion (from 1.54 mg g^−1^ to 0.81 mg g^−1^ of jelly serving). On the contrary, the progressive release of PGs from the chocolate matrix during the gastric and intestinal phases was achieved for jelly with the encapsulated extract. Although jelly disintegration began in the oral phase by hydrolysis of starch-based jelly shell, the lipid solubilization under bile salt presence in the intestinal fluid rapidly accelerated the process [103], thus completely releasing the HC from the analyzed jelly serving after 135 min (1.75 mg g^−1^). “Lipid coating” of CMC-based microparticles, as the result of its incorporation into the chocolate matrix, resulted in one form of the “layering“ and entrapment of encapsulates, enabling the protection of the extract within a delivery system from hydrophilic conditions and the slower release of targeted HC.

## 4. Conclusions

All CMC-based feed solutions exhibited pseudoplastic and viscous rather than elastic behavior. For the CMC blends, the decrease in apparent viscosity resulted in a higher process yield compared to the CMC used alone. In general, all CMC-based combinations proved to be very suitable to achieve high encapsulation efficiency for all analyzed PGs. High values of contact angles (105–144 °C) indicate strong cohesive forces between the microparticles and a lower wetting of the delivery systems, which obviously correlates positively with the prolonged in vitro release of the HC. ATR-FTIR analysis revealed the presence of hydrogen bonding between the polyphenolic structures and substitute units of the polymers. The incorporation of the CMC_CAR delivery system in the filling of jelly rolls proved to be an effective solution to achieve relatively high loadings of analyzed PGs (5–9 mg per dw serving). The application of CMC improved the physicochemical properties of the spray-dried microparticles enriched with the hydrophilic extract by increasing their hydrophobic properties and suitability for incorporation into the chocolate-based filling, thus allowing prolonged interaction between the food matrix used and the encapsulated extract in the simulated gastrointestinal environment.

As far as we know, this was the first time that kudzu starch was incorporated into the jelly matrix as a gelatin substitute. Considering the positive results of the sensory evaluation, further improvements could target the use of modified kudzu starch, with enhanced functional properties in terms of higher viscosity and improved pasting properties, freeze-thaw stability, and the retardation of starch retrogradation.

## Figures and Tables

**Table 1:** The recipes of formulated jelly rolls with corn and kudzu starch.

Ingredient	100CS	75CS_25K	50CS_50K	25CS_75K	100K
Sucrose (%)	58	58	58	58	58
Glucose syrup (%)	19	19	19	19	19
Corn starch (%)	19	14	9.5	5	/
Kudzu starch (%)	/	5	9.5	14	19
Citric acid (%)	0.9	0.9	0.9	0.9	0.9
Raspberry flavor (%)	0.4	0.4	0.4	0.4	0.4
Natural red color (%)	0.4	0.4	0.4	0.4	0.4
Lyophilized raspberries (%)	0.9	0.9	0.9	0.9	0.9
Glycerol (%)	1.4	1.4	1.4	1.4	1.4

**Table 2:** Determination of total hydroxycinnamic acids (HC) and individual phenylethanoid glycosides (PGs) of *T. montanum* extract.

HC (g L^−1^) *	Echinacoside (g L^−1^)	Teupolioside (g L^−1^) *	Stachysoside A (g L^−1^) *	Poliumoside (g L^−1^)	Verbascoside (g L^−1^)
mg g^−1^ dw of plant					
101.82 ± 3.66	23.54 ± 1.10	6.93 ± 0.42	13.39 ± 0.83	21.72 ± 0.22	7.90 ± 0.46
g L^−1^ of concentrated extract					
8.16 ± 0.00	2.38 ± 0.00	0.69 ± 0.00	1.30 ± 0.00	0.92 ± 0.00	0.60 ± 0.00

* g L^−1^-quantified as g of echinacoside equivalents per 1 L of extract.

**Table 3:** Values of storage modulus (G′), loss modulus (G″), loss factor (tan *δ*) at 1% shear strain, and apparent viscosity (*η*) at 25 s^−1^.

Sample	G′ _LVE_ (Pa)	G″ _LVE_ (Pa)	tan δ _LVE_	η (mPa·s)
CMC	2.28 ± 0.16 ^a^	3.95 ± 0.07 ^a^	1.75 ± 0.15	298.76 ± 1.05 ^a^
CMC_CLG	0.45 ± 0.08 ^a^	1.36 ± 0.06 ^ab^	3.16 ± 0.36	110.05 ± 0.66 ^a^
CMC_CAR	1.30 ± 0.32	2.44 ± 0.01 ^abc^	2.01 ± 0.51	168.86 ± 0.78 ^a^
CMC_AG	0.59 ± 0.13 ^a^	1.39 ± 0.12 ^ac^	2.44 ± 0.33	98.97 ± 0.22 ^a^
CMC_GG	0.32 ± 0.07 ^a^	1.01 ± 0.11 ^ac^	3.27 ± 0.36	72.08 ± 0.06 ^a^

CMC-carboxymethyl cellulose, CMC_CLG-carboxymethyl cellulose+collagen, CMC_CAR-carboxymethyl cellulose+κ-carrageenan, CMC_AG-carboxymethyl cellulose+gum arabic, CMC_GG-carboxymethyl cellulose+guar gum. Values marked with the same superscripted letter in the same column are statistically significant (*p* < 0.05).

**Table 4:** Physicochemical properties of CMC-based microparticles.

Samples	Process Yield (%)	Dry Matter (%)	Contact Angle (°)	Mean Particle Size (µm)	Zeta Potential (mV)	Polydispersity Index
CMC	32.55 ± 2.15	94.48 ± 0.92 ^a^	144 ± 1.00 ^a^	2.93 ± 1.46 ^a^	−15.59 ± 0.24 ^a^	0.95 ± 0.05 ^a^
CMC_CLG	54.48 ± 1.56	97.20 ± 0.11	105 ± 3.00 ^b^	3.09 ± 0.88 ^b^	−15.41 ± 0.48 ^a^	0.91 ± 0.09 ^a^
CMC_CAR	55.00 ± 2.01 ^a^	96.92 ± 0.34 ^a^	107 ± 1.50 ^b^	2.88 ± 1.23 ^abc^	−14.70 ± 0.35 ^a^	0.82 ± 0.04 ^a^
CMC_AG	53.16 ± 4.99 ^a^	95.94 ± 0.21 ^a^	140 ± 1.00 ^ac^	2.96 ± 1 13 ^bc^	−17.91 ± 0.48	1.00 ± 0.00 ^a^
CMC_GG	47.61 ± 3.56 ^a^	96.27 ± 0.05 ^a^	136 ± 2.87 ^ac^	2.39 ± 0.91 ^a^	−11.76 ± 0.35	0.97 ± 0.03 ^a^

CMC-carboxymethyl cellulose, CMC_CLG-carboxymethyl cellulose+collagen, CMC_CAR-carboxymethyl cellulose+κ-carrageenan, CMC_AG-carboxymethyl cellulose+gum arabic, CMC_GG-carboxymethyl cellulose+guar gum. Values marked with the same superscripted letter in the same column are not statistically different (*p* > 0.05).

**Table 5:** Encapsulation efficiency (%) and loading capacity (%) of HC and individual PGs of evaluated CMC-based microparticles.

	Encapsulation Efficiency (%)					
Sample	HC	Echinacoside	Teupolioside	Stachysoside A	Poliumoside	Verbascoside
CMC	76.85 ± 0.27	84.02 ± 0.20 ^a^	99.34 ± 0.16 ^ac^	88.13 ± 0.21	88.07 ± 0.77 ^a^	92.23 ± 0.83 ^a^
CMC_CLG	97.31 ± 0.31 ^a^	84.35 ± 0.16 ^a^	88.63 ± 0.38	83.79 ± 0.33	87.46 ± 0.15 ^a^	85.44 ± 0.07
CMC_CAR	68.67 ± 0.94	77.36 ± 0.50	80.41 ± 0.11	76.25 ± 0.19	79.51 ± 0.02	76.50 ± 0.25
CMC_AG	92.15 ± 2.57 ^ab^	90.62 ± 0.44 ^b^	103.31 ± 1.06 ^b^	94.07 ± 0.01	94.85 ± 0.20 ^b^	95.79 ± 0.49 ^b^
CMC_GG	90.12 ± 1.07 ^ab^	90.61 ± 0.42 ^b^	101.66 ± 0.07 ^abc^	92.71 ± 0.01	94.04 ± 0.02 ^b^	94.80 ± 0.32 ^ab^
	Loading capacity (%)					
CMC	20.15 ± 0.40	6.13 ± 0.01	1.80 ± 0.03	3.32 ± 0.02	2.37 ± 0.06	1.55 ± 0.01
CMC_CLG	23.12 ± 0.36	4.54 ± 0.01 ^a^	1.17 ± 0.02	2.33 ± 0.00	1.73 ± 0.01 ^a^	1.09 ± 0.01 ^a^
CMC_CAR	22.30 ± 0.38	4.37 ± 0.02 ^a^	1.10 ± 0.01	2.17 ± 0.01	1.65 ± 0.01 ^a^	0.99 ± 0.00 ^a^
CMC_AG	22.09 ± 0.08	4.10 ± 0.01 ^a^	1.18 ± 0.02	2.20 ± 0.01	1.62 ± 0.01 ^a^	1.01 ± 0.01 ^a^
CMC_GG	21.55 ± 0.45	4.21 ± 0.02 ^a^	1.20 ± 0.00	2.21 ± 0.02	1.61 ± 0.03 ^a^	1.04 ± 0.01 ^a^

HC-total hydroxycinnamic acids derivatives; CMC-carboxymethyl cellulose, CMC_CLG-carboxymethyl cellulose+collagen, CMC_CAR-carboxymethyl cellulose+κ-carrageenan, CMC_AG-carboxymethyl cellulose+gum arabic, CMC_GG-carboxymethyl cellulose+guar gum. Values marked with the same superscripted letter in the same column are not statistically different (*p* > 0.05).

**Table 6:** HPLC determination of phenylethanoid glycosides and methylxanthines in formulated jellies.

Sample	mg g^−1^ per Serving						
	Echinacoside	Teupolioside *	Stachysoside A *	Poliumoside *	Verbascoside	Teobromine	Caffeine
100CS	2.27 ± 0.06	0.80 ± 0.01	0.94 ± 0.02	2.99 ± 0.07	0.85 ± 0.02	4.99 ± 0.11	0.54 ± 0.01
75CS_25K	1.38 ± 0.01	0.47 ± 0.00	0.56 ± 0.00	1.81 ± 0.02	0.50 ± ±0.01	3.11 ± 0.03	0.33 ± 0.00
50CS_50K	2.12 ± 0.03	0.70 ± 0.00	0.86 ± 0.02	2.71 ± 0.04	0.770.01	4.60 ± 0.06	0.49 ± 0.01
25CS_75K	2.29 ± 0.02	0.76 ± 0.00	0.92 ± 0.01	2.91 ± 0.03	0.82 ± 0.01	4.92 ± 0.04	0.53 ± 0.01
100K	2.73 ± 0.00	0.92 ± 0.01	1.12 ± 0.01	3.48 ± 0.00	1.01 ± 0.00	5.83 ± 0.01	0.62 ± 0.00

CS—corn starch; K—kudzu starch; * quantified as mg echinacoside equivalents per dry weight of one candy serving (1 serving = 6 g).

**Table 7:** Textural properties of formulated candy samples measured by TPA test.

Sample	Hardness (g)	Cohesiveness	Springiness	Chewiness	Resilience	Adhesiveness (g.s)
100CS	794.94 ± 7.42 ^a^	1.22 ± 0.67	1970.45 ± 370.45	35,506.30 ± 1428	32.29 ± 5.88	−5.04 ± 1.26
75CS_25K	850.78 ± 9.79	1.67 ± 0.14	2305.68 ± 26.14	32,855.89 ± 538	37.15 ± 1.79	−10.16 ± 3.85
50CS_50K	765.81 ± 14.40 ^b^	1.91 ± 0.11	2327.27 ± 4.55	34,089.32 ± 1312	35.55 ± 0.63	−6.66 ± 0.78
25CS_75K	829.46 ± 62.90	1.38 ± 0.45	2248.86 ± 87.50	25,446.69 ± 1408	34.02 ± 1.39	−10.89 ± 6.64
100K	978.94 ± 28.42 ^ab^	1.63 ± 0.03	2359.09 ± 2.27	37,701.42 ± 425	38.17 ± 5.20	−3.03 ± 2.19

CS—corn starch; K—kudzu starch; values marked with the same superscripted letter in the same column are statistically different (*p* < 0.05).

## Data Availability

The research data are provided within the manuscript. The raw data supporting the results of this study are available on request from the corresponding author.
